# First multicenter real-world analysis of switching to next-generation enzyme replacement therapies in late-onset Pompe disease

**DOI:** 10.1007/s00415-026-13689-1

**Published:** 2026-02-14

**Authors:** Daniel H. Mendelsohn, Angela Rosenbohm, Anne-Katrin Güttsches, Cornelia Kornblum, Karl Christian Knop, Tanja Fangerau, Nam Nguyen-Younossi, Guljan Shahyrova, Natalia Garcia-Angarita, Benedikt Schoser, Stephan Wenninger

**Affiliations:** 1 Friedrich-Baur Institute at the Department of Neurology, LMU Clinic Munich, Ziemssenstr.1, 80336 Munich, Germany; 2 Department of Neurology, Ulm University Clinic, 89081 Ulm, Germany; 3 Department of Neurology, Heimer Institute for Muscle Research, BG-University Hospital Bergmannsheil gGmbH, Ruhr-University Bochum, Bochum, Germany; 4 Heimer Institute for Muscle Research, BG-University Hospital Bergmannsheil gGmbH, Bochum, Germany; 5 Center for Neurology, Department of Neuromuscular Diseases, University Hospital Bonn, Venusberg-Campus 1, 53127 Bonn, Germany; 6 Neurologie Neuer Wall, 20354 Hamburg, Germany

**Keywords:** Late-onset Pompe disease, Enzyme replacement therapy, Real-world evidence, Treatment switch, Neuromuscular disorders

## Abstract

**Background:**

Next-generation enzyme replacement therapies (ERTs) for late-onset Pompe disease (LOPD), including avalglucosidase alfa and cipaglucosidase alfa with miglustat, have been developed to improve muscle targeting and enzyme stability. Real-world evidence on therapy switching between ERT preparations remains limited.

**Methods:**

A prospective, observational, multicenter cohort study was conducted across German neuromuscular centers. Adults with genetically confirmed LOPD who transitioned to avalglucosidase alfa (Aval) or cipaglucosidase alfa with miglustat (Cipa/Mig) between August/2022 and September/2024 were included. Clinical data were extracted from medical records using a standardized case report form. Following EPOC recommendations, data captured comprised six-minute walk test (6MWT), ten-meter walk test (10MWT), Rasch-built Pompe-specific Activity scale (R-PAct), upright and supine forced vital capacity (FVC), maximal inspiratory/expiratory pressure (MIP/MEP), and maximal voluntary ventilation (MVV). Data were analyzed descriptively and using linear mixed-effects models. Paired comparisons between baseline and 12 months were performed as a secondary analysis in patients with available data.

**Results:**

Thirty-nine patients (43 switches; alglucosidase alfa (AlGlu) → Aval *n* = 32, AlGlu → Cipa/Mig *n* = 7) were included; four double switches were analyzed under AlGlu → Aval. Main reasons for switching were patient request (42%) and clinical worsening (40%). Overall data completeness declined over time. Mean trajectories indicated stable respiratory function, minimal change in R-PAct, and non-significant trends toward slower walking performance. Mixed-model analyses revealed no significant effects of time or switch type, with baseline performance as the only consistent predictor. Infusion-related or serious adverse events were rare, and EPOC documentation criteria were met in 56% of patients.

**Conclusion:**

This real-world study suggests that transitions between ERT preparations are generally feasible and associated with clinical stability in LOPD. Switching may represent a useful strategy in patients, particularly when efficacy concerns arise. Standardized prospective studies with systematic monitoring of immunogenicity and efficacy according to the 2024 EOPC guideline are recommended to confirm these findings.

**Supplementary Information:**

The online version contains supplementary material available at 10.1007/s00415-026-13689-1.

## Introduction

Late-onset Pompe disease (LOPD) is an autosomal recessive lysosomal storage disorder caused by pathogenic variants in both alleles of the *GAA* gene, resulting in deficiency of acid α-glucosidase and progressive lysosomal glycogen accumulation. In adults, the disease predominantly affects skeletal and respiratory muscles, manifesting as slowly progressive proximal and axial weakness and restrictive ventilatory failure due to predominantly diaphragmatic involvement [1, 2]. Without treatment, LOPD typically follows a gradual but progressive course, with measurable decline in functional parameters such as forced vital capacity (FVC), six-minute walk test (6MWT), and timed walking assessments [3]. Respiratory insufficiency remains a leading cause of morbidity and mortality, highlighting the need for disease-modifying interventions [4].

ERT with recombinant human acid α-glucosidase (alglucosidase alfa) has been the standard of care since its approval in 2006, improving survival and attenuating disease progression [5, 6]. A recent UK review further confirmed the superiority of ERT over best supportive care, while noting substantial limitations in real-world practice, including poor adherence to monitoring protocols and limited long-term outcome data. Nevertheless, significant limitations remain, including suboptimal skeletal muscle uptake due to restricted mannose-6-phosphate receptor-mediated delivery and infusion-associated reactions, which in severe cases may necessitate treatment modification or discontinuation [7]. To address these shortcomings, next-generation therapies have been developed. Avalglucosidase alfa was engineered with increased mannose-6-phosphate residues to enhance muscle targeting and has demonstrated improved pharmacokinetics and clinical outcomes in pivotal trials. Cipaglucosidase alfa, co-administered with the oral pharmacological enzyme stabilizer miglustat, alleviates the enzyme and increases systemic bioavailability, offering an alternative therapeutic option [8, 9].

Both agents have demonstrated efficacy and acceptable safety in phase-3 trials. In the COMET study, avalglucosidase alfa showed non-inferiority to alglucosidase alfa in ambulatory LOPD patients, with trends toward greater improvement in respiratory and functional outcomes [6]. In the PROPEL trial, cipaglucosidase alfa plus miglustat was associated with clinically meaningful gains in functional endurance and respiratory measures compared with alglucosidase alfa plus placebo [7]. These results supported European Medicines Agency approval of avalglucosidase alfa in 2022 and cipaglucosidase alfa with miglustat in 2023. However, trial populations are typically highly selected, with strict inclusion criteria and controlled infusion protocols, and may not reflect the heterogeneity, comorbidities, and treatment patterns encountered in routine care.

Recognizing the emergence of multiple ERT options, the European POmpe Consortium (EPOC) updated its recommendations in 2024, introducing “triple-S” criteria (start, switch, stop) to guide therapy decisions [8, 9]. Switching to an alternative ERT is advised when skeletal and/or respiratory function fails to stabilize or improve after ≥ 12 months of standard therapy, or in the event of severe, unmanageable IARs. While phase-3 data support the efficacy of both novel agents, there is limited real-world evidence on outcomes after switching from established ERT or between novel agents, and on the extent to which EPOC criteria are implemented in clinical practice.

The present study aimed to evaluate the efficacy and safety of avalglucosidase alfa and cipaglucosidase alfa with miglustat in a German-wide multicenter cohort of LOPD patients transitioning to these treatments. In addition, we assessed adherence to the updated EPOC switching criteria in a real-world setting.

## Methods

We conducted a prospective, observational, multicenter cohort study across specialized neuromuscular centers in Germany with long-standing expertise in the diagnosis and treatment of late-onset Pompe disease (LOPD). Eligible patients were adults (≥ 18 years) with genetically confirmed LOPD, defined by pathogenic variants in both alleles of the GAA gene and reduced acid α-glucosidase activity. Inclusion required a transition to either avalglucosidase alfa or cipaglucosidase alfa with miglustat, irrespective of the previous enzyme replacement therapy (ERT) regimen, with available clinical and functional assessments within 6 months before the switch and at least one follow-up evaluation. Patients were excluded if they participated in blinded interventional clinical trials during the observation period.

Switches comprised transitions from alglucosidase alfa to avalglucosidase alfa (AlGlu → Aval) or cipaglucosidase alfa with miglustat (AlGlu → Cipa). Four patients underwent double switches but were analyzed within the AlGlu → Aval group, as no follow-up data were available beyond the second switch.

Data were extracted from medical records and institutional Pompe disease registries on June 1, 2025, using a standardized case report form. Baseline values were defined as the most recent assessments within 6 months before the switch. Follow-up data were collected at approximately 6, 12, 18, and 24 months, when available.

The main diagnostic assessments included the Rasch-built Pompe-specific Activity scale (R-PAct), six-minute walk test (6MWT), ten-meter walk test (10mWT), forced vital capacity (FVC) in upright and supine positions, maximal inspiratory pressure (MIP), maximal expiratory pressure (MEP), and maximal voluntary ventilation (MVV). Safety data comprised infusion-associated reactions (IARs), adverse and serious adverse events (AEs/SAEs), and anti-drug antibody (ADA) titers when available. The clinical rationale for switching was extracted from patient charts and categorized according to common clinical practice (disease progression, patient request, adverse events). Adherence to the EPOC switch recommendations was assessed by determining, for each patient, whether at least one functional parameter (6MWT, 10mWT, WGMS, GSGC, QMFT), one respiratory parameter (upright or supine FVC, MVV, MIP, MEP), and one patient-reported outcome (R-PAct) had been documented before the switch. The proportion of patients fulfilling these criteria was calculated to quantify adherence.

For statistical analysis, continuous outcomes were evaluated using linear mixed-effects models with time (baseline, 6, 12, 18, 24 months) as the within-subject factor. Switch type (AlGlu → Aval vs. AlGlu → Cipa) was included as a between-subject factor. Baseline performance, gender, and disease duration were entered as covariates. Estimated marginal means (EMMs) and pairwise comparisons were reported. As a secondary analysis, paired comparisons between baseline and 12-month follow-up were conducted in patients with available data at both time points. Paired two-sided *t* tests were applied to assess intra-individual changes in 6MWT, 10mWT, and upright FVC (% predicted). Analyses were performed using SPSS (version 29; IBM Corp., Armonk, NY, USA). A two-sided *p* value < 0.05 was considered statistically significant. The study was conducted in accordance with the Declaration of Helsinki. The study was approved by the ethics committee of the LMU clinic, project No. 25–0410.

## Results

**Fig. 1:**
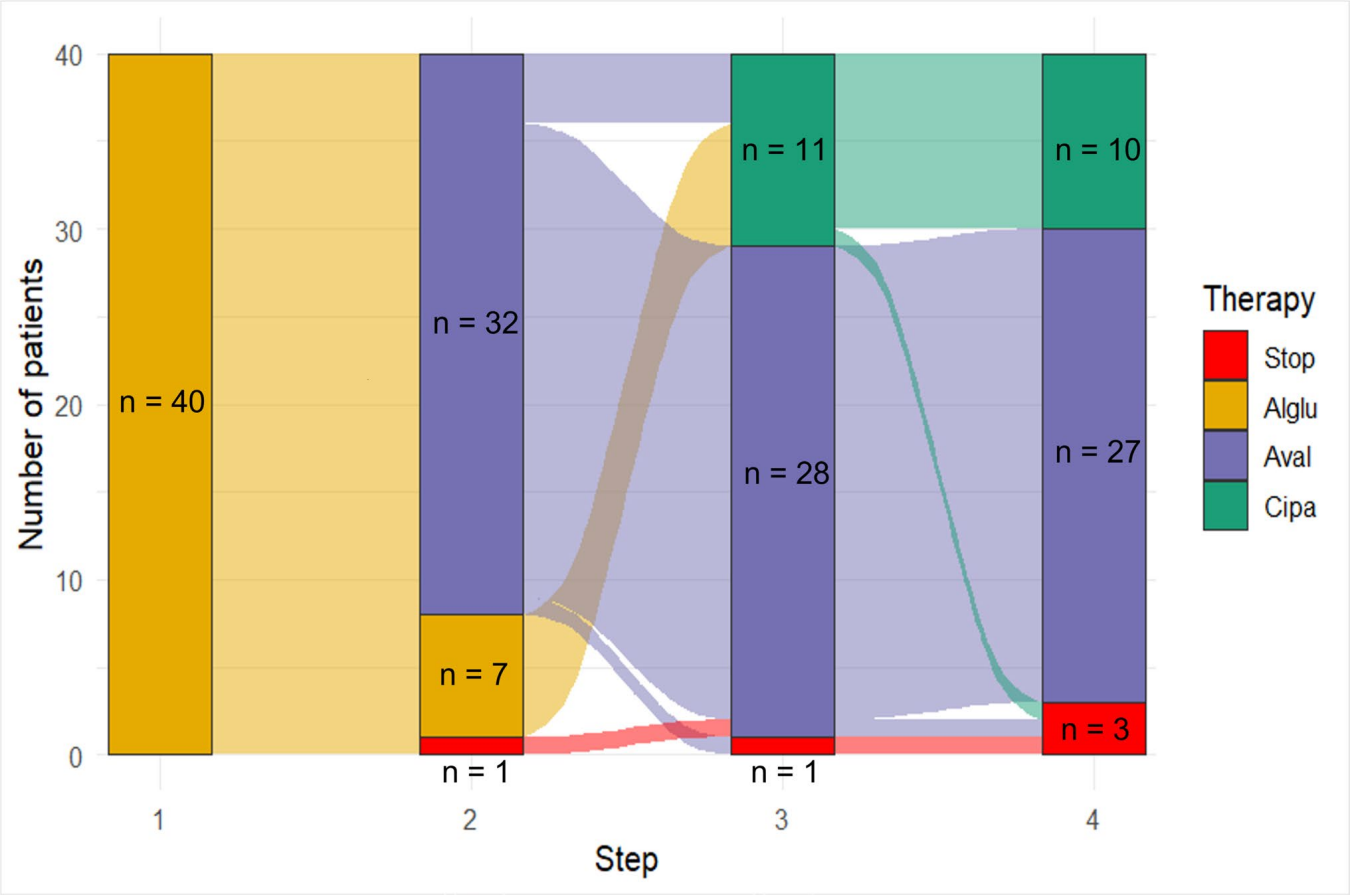
Alluvial plot of therapy transitions in Pompe disease. Four analytic steps denote each patient’s ordered therapy states (not synchronized calendar time; switches occurred at different real-world times). Bar segments indicate therapy categories present at each step, and flows depict transitions between therapies. Bar segments are annotated with n = patients per step and therapy. Alglu, alglucosidase alfa; Aval, avalglucosidase alfa; Cipa, cipaglucosidase alfa + miglustat; Stop, therapy discontinuation

A total of 39 patients from 5 participating sites were included in the analysis (25 females, 14 males): the Department of Neurology, University Hospital Bergmannsheil, Bochum; the Department of Neuromuscular Diseases, University Hospital Bonn; Neurology Practice Hamburg Neuer Wall; the Department of Neurology, University Hospital Ulm; and the Friedrich-Baur-Institute, LMU Munich (all in Germany). The majority of patients (*n* = 32) transitioned from alglucosidase alfa to avalglucosidase alfa (AlGlu → Aval), while seven patients switched from alglucosidase alfa to cipaglucosidase alfa (AlGlu → Cipa). Four patients underwent double switches (Fig. 1). However, they were analyzed in the AlGlu → Aval group as no follow-up data were available beyond the second switch. In total, 43 individual switches were documented. The most frequently reported reasons for switching were patient request (18/43) and continuous clinical worsening (17/43). Two switches were performed due to adverse events, while in six patients, no reason was documented.

The number of diagnostic assessments decreased over time across all outcome measures (Table 1). For the 6MWT, 29 patients were assessed before the switch, 22 at 6 months, and 10 at 24 months. Similarly, the number of 10mWT assessments declined from 31 at baseline to 20 at 6 months and 10 at 24 months. For respiratory parameters, upright FVC was available in 33 patients at baseline and in 11 at 24 months, while supine FVC decreased from 17 to 10 patients. MVV was performed at baseline in 13 patients, but only 2 at 24 months. Data availability for MIP and MEP also declined, from 12 and 11 patients at baseline to 4 patients at 24 months, respectively. Motor function scales showed a similar pattern, with QMFT decreasing from 32 at baseline to 12 at 24 months, and GSGC from 14 to 6. The patient-reported outcome R-PAct was completed by 24 patients at baseline and 10 at 24 months. Finally, anti-drug antibody (ADA) testing was available in only 16 patients before the switch and in only 2–4 patients during follow-up. Overall, data availability declined markedly across all outcome domains, substantially limiting the statistical power of longitudinal analyses. To evaluate adherence to EPOC recommendations, we determined the proportion of patients with documentation of at least one functional, one respiratory, and one patient-reported outcome measure before switching. This criterion was met in 22 of 39 patients, yielding an adherence rate of 56.4%.

**Table 1:** Number of patients with available assessments at each follow-up time point

Test	Pre-switch	6 months	12 months	18 months	24 months
6MWT	29	22	15	11	10
10mWT	31	20	15	11	10
FVC (%) upright	33	22	15	15	11
FVC (%) supine	17	14	10	12	10
MVV	13	3	5	4	2
MIP	12	4	4	4	4
MEP	11	4	4	4	4
QMFT	32	20	15	14	12
GSGC	14	11	9	10	6
R-Pact	24	17	15	13	10
ADA	16	2	4	2	3

6MWT, six-minute walk test; 10mWT, 10-m walk test; FVC, forced vital capacity; MVV, maximal voluntary ventilation; MIP, maximal inspiratory pressure; MEP, maximal expiratory pressure; QMFT, quick motor function test; GSGC, Gait, Stairs, Gower, Chair test; R-Pact, Rasch-built Pompe-specific Activity scale; ADA, anti-drug antibodies

**Fig. 2:**
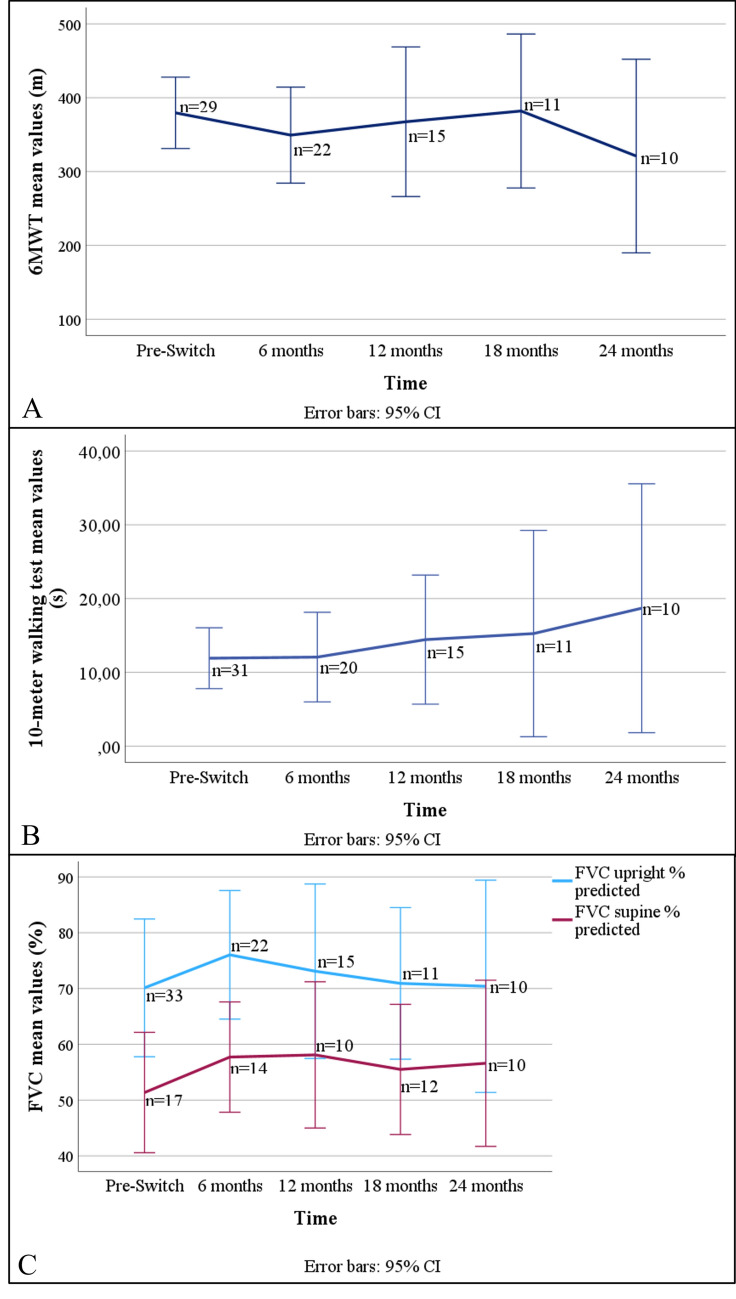
Longitudinal changes in (A) 6MWT, (B) 10mWT, and (C) upright and supine FVC (% predicted) after ERT switching in LOPD. Data shown as means ± 95% CI; n indicates patients per time point

Across the cohort, mean FVC% upright remained largely stable over the 24-month follow-up (baseline 67.7% ± 21.9; 24 months 72.5% ± 26.2; Δ + 4.7%). Supine FVC% showed a similar pattern (baseline 51.4% ± 21.0; 24 months 56.6% ± 20.8; Δ + 5.3%). R-PAct scores varied minimally (baseline 21.2 ± 7.9; 24 months 22.5 ± 8.7; Δ + 1.3). In contrast, mean 10mWT times increased from 11.9 ± 11.2 s at baseline to 18.7 ± 23.6 s at 24 months (Δ + 6.8 s). Mean 6MWT distance declined from 379.5 ± 127.1 m at baseline to 321.0 ± 183.3 m at 24 months (Δ –58.5 m) (Fig. 2).

In the mixed-model analyses, none of the tested outcomes showed significant effects of time, switch type, or their interaction (Table 2). For the 6-min walk test (6MWT), baseline performance was the only significant predictor of longitudinal outcomes (*p* < 0.001). In contrast, no significant effects of time (*p* = 0.724), switch type (*p* = 0.370), or their interaction (*p* = 0.522) were observed. Estimated marginal means (EMMs) for the overall cohort indicated a modest decline, with mean changes of –20 m at 6–12 months and up to –32 m at 18 months relative to pre-switch. However, these differences did not reach statistical significance.

**Table 2:** Results of linear mixed model analyses for functional (6MWT, 10mWT) and respiratory (upright FVC%) outcomes. Shown are numerator and denominator degrees of freedom (df), F-statistics, and p values from Type III tests of fixed effects. None of the outcomes demonstrated significant main effects of time, switch type, or their interaction. Among the covariates, baseline values of the respective outcome measures (6MWT, 10mWT, FVC upright) were significant predictors, while gender and disease duration did not show significant effects

Outcome	Effect	df (num, den)	F	*p* value
6MWT	Switch type	1, 39.98	0,823	0,37
	Time	4, 48.53	0,517	0,724
	Time × switch type	4, 42.93	0,417	0,522
	Disease duration	1, 20.65	0,161	0,692
	6MWT baseline	1, 18.95	400,298	< 0.001
	Gender	1, 18.26	1,449	0,244
10mWT	Switch type	1, 26.14	0,082	0,777
	Time	4, 51.44	0,791	0,537
	Time × switch type	–		
	Disease duration	1, 22.97	0,32	0,577
	10mWT baseline	1, 36.54	88,989	< 0.001
	Gender	1, 22.90	0,988	0,331
FVC (%) upright	Switch type	1, 29.64	0,701	0,409
	Time	4, 51.84	1,642	0,178
	Time × switch type	4, 51.84	1,782	0,146
	Disease duration	1, 27.77	1,24	0,275
	FVC upright baseline	1, 49.50	962,177	< 0.001
	Gender	1, 27.87	0,449	0,508

Similarly, for the 10-m walk test (10mWT), neither time (*p* = 0.537) nor switch type (*p* = 0.777) showed significant effects, and the interaction could not be reliably estimated. EMMs suggested a gradual, non-significant increase in walking time, from 9.5 s at baseline to 16.2 s at 24 months (Table S1, Supplementary Material).

For upright FVC%, no significant effects of time (*p* = 0.178), switch type (*p* = 0.409), or their interaction (*p* = 0.146) were detected. EMMs remained relatively stable over the 24-month observation period (71.7% predicted at baseline vs. 74.5% at 24 months; Δ + 2.8%).

Across all outcomes, pairwise comparisons did not reveal any significant differences between time points. Group comparisons between switch types were further limited by data scarcity, particularly in the AlGlu → Cipa subgroup.

As a secondary analysis, paired comparisons between baseline and 12 months were performed in patients with available data at both time points (*n* = 14). These analyses showed no statistically significant intra-individual changes in functional or respiratory outcomes. Mean 6MWT distance declined by 16.43 m (*p* = 0.305), 10mWT time increased by 3.83 s (*p* = 0.145), and FVC (%) upright changed minimally, with a mean difference of − 1.1% predicted (*p* = 0.621). These findings were consistent with the mixed-model results, supporting overall clinical stability following ERT switching (Table 3).

**Table 3:** Paired secondary analysis of functional and respiratory outcomes between baseline and 12 months after ERT switching. Values are shown as mean ± standard deviation. Mean change was calculated as 12 months minus baseline. Paired two-sided *t* tests were used

Outcome	n	Baseline mean ± SD	12-month mean ± SD	Mean change ± SD		*p* value	
6MWT	14	383.86 (± 141.29)	367.43 (± 175.49)		−16.43 (± 57.43)		0.305
10mWT	14	10.61 (± 6.78)	14.43 (± 15.79)		+ 3.83 (± 9.61)		0.145
FVC (%) upright	14	69.95 (± 22.06)	68.89 (± 22.22)		−1.06 (± 7.86)		0.621

6MWT, six-minute walk test; 10mWT, ten-meter walk test; FVC, forced vital capacity

During follow-up, infusion-associated reactions (IARs) were infrequent, occurring in 2/39 patients at baseline, 3/39 at 6 months, 1/23 at 18 months, and none thereafter (Table 4). Serious adverse events (SAEs) were rare, with 2/39 patients affected at 6 months and no further events observed during subsequent follow-up.

**Table 4:** Frequency of infusion-associated reactions (IAR) and serious adverse events (SAE) at baseline and during follow-up after ERT switching in LOPD. Values indicate number of patients with events per total assessed at each time point

	Pre-switch	6 months	12 months	18 months	24 months
IAR	2/39	3/39	0/28	1/23	0/13
SAE	0/39	2/39	0/39	0/21	0/13

Regarding functional status, 18/40 patients were able to walk unassisted at baseline and 5/13 at 24 months, while the frequency of nocturnal non-invasive ventilation (NIV) use remained relatively stable, decreasing from 19/39 to 6/13 over the same period (Table 5).

**Table 5:** Frequency of patients walking unassisted and using non-invasive ventilation (NIV) before and after ERT switching in LOPD. Values indicate the number of patients with the respective status per total assessed at each time point

	Pre-switch	6 months	12 months	18 months	24 months
Walking unassisted	18/40	18/37	11/28	11/22	5/13
NIV	19/39	19/37	14/27	11/22	6/13

## Discussion

This is the first multicenter, prospective study to provide real-world data on treatment switches among alglucosidase alfa, avalglucosidase alfa, and cipaglucosidase alfa in patients with late-onset Pompe disease (LOPD). Despite heterogeneity in available assessments, the cohort remained clinically stable after transition, without any occurrences or increases in AE/SAEs or IARs, supporting the notion that both next-generation ERTs represent safe and effective therapeutic options in routine practice. Reviews of the phase-3 data highlight the superiority of next-generation ERTs over alglucosidase alfa, while emphasizing that confirmation of these benefits in real-world settings remains limited [8].

The overall stability observed in this cohort contrasts with the modest functional gains reported in pivotal clinical trials. In the COMET study, avalglucosidase alfa demonstrated non-inferiority to alglucosidase alfa with trends toward greater improvements in respiratory and motor outcomes. In the PROPEL trial, cipaglucosidase alfa plus miglustat was associated with clinically meaningful improvements in functional endurance and lung function [6, 7].

Differences between these results and our findings likely reflect the highly selected nature of trial populations, standardized infusion protocols, and rigorous follow-up schedules, compared with the heterogeneity and comorbidities encountered in routine care in the real-world setting.

Interpretation of immunological findings, including anti-drug antibody (ADA) development, was not possible in our analysis due to the limited and inconsistent availability of relevant data across centers and the incomparability of the two company-based assays used to measure anti-drug antibodies. More systematic monitoring of immunogenicity will be important in future studies to clarify its impact on long-term safety and efficacy of next-generation ERT. Within these constraints, the findings suggest that therapy switching may be a valuable strategy even when products are considered therapeutically equivalent. For some patients, switching could mitigate tolerability issues, such as infusion-associated reactions, while preserving clinical stability. This observation is consistent with previous reports describing improved tolerability after transitions between different ERT preparations [6, 7].

Several methodological limitations must be considered. Follow-up data were incomplete—particularly beyond 12 months—reducing statistical power and limiting the interpretability of long-term outcomes. Owing to the retrospective design, diagnostic and functional assessments were not standardized across centers, with variable adherence to EPOC-recommended outcome measures, thereby introducing heterogeneity and complicating between-center comparisons. Finally, as a prospective real-world study, the analysis is subject to inherent selection and reporting biases. Collectively, these limitations underscore the ongoing need for long-term prospective, standardized data collection with systematic follow-up and immunogenicity monitoring to validate these observations and to identify patient subgroups most likely to benefit from a therapy switch.

## Conclusion

In this multicenter real-world analysis, switching between ERT preparations in LOPD was feasible and associated with overall clinical stability. However, interpretation of outcomes was markedly limited by scarce and incomplete follow-up data, reflecting the challenges of retrospective, multicenter research in rare diseases. Continuous and systematic long-term data collection through dedicated patient registries will be crucial to overcoming these limitations. Addressing this gap through prospective studies with standardized assessments and systematic long-term follow-up will be essential to define outcomes better and identify patient subgroups most likely to benefit from therapy switching.

## Supplementary Information

Below is the link to the electronic supplementary material.

Supplementary file1 (DOCX 18 KB)

## Data Availability

The data that support the fi ndings of this study are available from the corresponding author upon reasonable request.
