# Supplementary material for: First multicenter real-world analysis of switching to next-generation enzyme replacement therapies in late-onset Pompe disease
Source: J Neurol. 2026 Feb 14;273(2):144. doi: 10.1007/s00415-026-13689-1 (PMC12906561; doi:10.1007/s00415-026-13689-1)
Supplement: Supplementary file 1 — Supplementary file1 (DOCX 18 KB) [file 415_2026_13689_MOESM1_ESM.docx]

**First Multicenter Real-World Analysis of Switching to Next-Generation Enzyme Replacement Therapies in Late-Onset Pompe Disease**Daniel H. Mendelsohn^1^, Angela Rosenbohm^2^, Anne-Katrin Güttsches^3,4^, Cornelia Kornblum^5^, Karl Christian Knop^6^, Tanja Fangerau^2^, Nam Nguyen-Younossi^2^, Guljan Shayrova^1^, Natalia Garcia-Angarita^1^, Benedikt Schoser^1^, Stephan Wenninger^1,*^

^1^ Friedrich-Baur Institute at the Department of Neurology, LMU Clinic Munich, Ziemssenstr.1, 80336 Munich, Germany
^2^ Department of Neurology, Ulm University Clinic, 89081 Ulm, Germany
^3^ Department of Neurology, Heimer Institute for Muscle Research, BG-University Hospital Bergmannsheil gGmbH, Ruhr-University Bochum, Bochum, Germany
^4^ Heimer Institute for Muscle Research, BG-University Hospital Bergmannsheil gGmbH, Bochum, Germany
^5^ Center for Neurology, Department of Neuromuscular Diseases, University Hospital Bonn, Venusberg-Campus 1, 53127 Bonn, Germany
^6^ Neurologie Neuer Wall, 20354 Hamburg, Germany

^*^Corresponding author: stephan.wenninger@med.uni-muenchen.de

**Supplementary material**

| **Time point** | **6MWT (m)** | **10mWT (s)** | **FVC (%) upright** |
| --- | --- | --- | --- |
| Baseline | 387.1 (±22.3) | 9.51 (±8.09) | 71.68 (±7.31) |
| 6 months | 367.2 (±9.3) | 13.11 (±3.13) | 71.28 (±7.44) |
| 12 months | 367.0 (±10.7) | 14.55 (±3.23) | 76.27 (±7.83) |
| 18 months | 354.8 (±12.0) | 14.96 (±3.39) | 70.00 (±7.95) |
| 24 months | 359.0 (±22.9) | 16.20 (±3.58) | 74.52 (±7.83) |

**Tab. S1:** Estimated marginal means (EMMs ± standard error) for 6MWT, 10mWT, and upright FVC% at baseline and follow-up time points (6, 12, 18, and 24 months). Values were adjusted for baseline performance, gender, and disease duration. Across all outcomes, changes over time did not reach statistical significance, and no consistent group differences were observed.
